# Representing Kidney Development Using the Gene Ontology

**DOI:** 10.1371/journal.pone.0099864

**Published:** 2014-06-18

**Authors:** Yasmin Alam-Faruque, David P. Hill, Emily C. Dimmer, Midori A. Harris, Rebecca E. Foulger, Susan Tweedie, Helen Attrill, Douglas G. Howe, Stephen Randall Thomas, Duncan Davidson, Adrian S. Woolf, Judith A. Blake, Christopher J. Mungall, Claire O’Donovan, Rolf Apweiler, Rachael P. Huntley

**Affiliations:** 1 European Molecular Biology Laboratory, European Bioinformatics Institute (EMBL-EBI), Wellcome Trust Genome Campus, Hinxton, Cambridge, United Kingdom; 2 The Jackson Laboratory, Bar Harbor, Maine, United States of America; 3 Cambridge Systems Biology Centre and Department of Biochemistry, Sanger Building, University of Cambridge, Cambridge, United Kingdom; 4 FlyBase, Department of Genetics, University of Cambridge, Cambridge, United Kingdom; 5 The Zebrafish Model Organism Database (ZFIN), University of Oregon Eugene, Oregon, United States of America; 6 IR4M UMR8081, Centre National de la Recherche Scientifique (CNRS), Université Paris-Sud, Orsay, Franc; 7 MRC Human Genetics Unit, Institute of Genetics and Molecular Medicine, Western General Hospital, Edinburgh, United Kingdom; 8 Institute of Human Development, Faculty of Medical and Human Sciences, University of Manchester, Manchester, United Kingdom; 9 Genomics Division, Lawrence Berkeley National Laboratory, Berkeley, California, United States of America; UCL Institute of Child Health, United Kingdom

## Abstract

Gene Ontology (GO) provides dynamic controlled vocabularies to aid in the description of the functional biological attributes and subcellular locations of gene products from all taxonomic groups (www.geneontology.org). Here we describe collaboration between the renal biomedical research community and the GO Consortium to improve the quality and quantity of GO terms describing renal development. In the associated annotation activity, the new and revised terms were associated with gene products involved in renal development and function. This project resulted in a total of 522 GO terms being added to the ontology and the creation of approximately 9,600 kidney-related GO term associations to 940 UniProt Knowledgebase (UniProtKB) entries, covering 66 taxonomic groups. We demonstrate the impact of these improvements on the interpretation of GO term analyses performed on genes differentially expressed in kidney glomeruli affected by diabetic nephropathy. In summary, we have produced a resource that can be utilized in the interpretation of data from small- and large-scale experiments investigating molecular mechanisms of kidney function and development and thereby help towards alleviating renal disease.

## Introduction

All complex organisms require the ability to balance fluids and excrete toxic metabolic byproducts. Renal systems achieve this by filtering and excreting substances using specialized cells, tissues and organs. As researchers have embraced proteomic and genomic investigative methods to identify, quantify and characterize pathways and networks associated with the renal system over the past decade, a wealth of biological information has resulted [1]–[8]. This data deluge is often time-consuming for researchers to analyse, and highlights the need for a representation of renal biology that enables high-quality, detailed, computational analysis. Given that renal researchers make extensive use of model organisms, such a resource needs to take account of the similarities and differences between species in order to provide a species-neutral representation of development and allow for cross-species comparison. Although the renal system is generally composed of tubules that transport water and solutes between an organism and its external environment, the system structure differs across species. In insects, and some other classes, the renal system is comprised of Malpighian tubules, whereas in vertebrates and some invertebrates it is made up of several organs, with the kidney being the main player in fluid and solute exchange. While renal systems differ in structure throughout the animal kingdom, there are necessary physiological similarities [9]. These physiological processes must be represented in a general way to allow effective comparisons between species. In addition, the resource needs to provide for the nomenclature differences that arise. Even with the existence of a standard nomenclature for structures of the kidney proposed by the Renal Commission of the International Union of Physiological Sciences [10] and a high-resolution ontology to describe the sub-compartments of the developing murine genitourinary tract developed by the GUDMAP Consortium [11], there is still linguistic ambiguity amongst the renal community regarding the naming of processes associated with the function and development of the renal system. For example, ‘nephrogenesis’ is used by some to refer to the process of overall kidney development, but is also commonly used to describe the formation of the individual functioning nephrons within the kidney.

The Gene Ontology (GO) project aims to provide a structured vocabulary that can be used to annotate gene products from any species in the context of their role within an organism and their location within a cell or in the vicinity of a cell. We embarked on a project to improve the way in which GO describes the processes of renal development and physiology [12]. GO terms referencing renal anatomical structures were made consistent with existing resources including the GUDMAP Consortium [8], the Cell Type Ontology [13] and the multi-species Uber anatomy ontology (UBERON) [14]. Additionally, cross-references [15] were created between renal system processes in GO and anatomical structures in UBERON. In doing so, we provide a framework wherein additional renal-related terms may be added in the future.

To utilize the expanded ontology, renal- and non renal-related GO terms were associated with gene products involved in renal development. These annotations were established initially through the process of manual curation, in which a curator reviewed the primary literature for experimental evidence to create a gene product-GO association (annotation). Secondly, where appropriate, these experimentally inferred annotations were transferred to equivalent gene products in other species [16]–[18]. This transfer was performed both manually, by a curator following a BLAST sequence similarity search [19] and electronically, via the Ensembl Compara automatic annotation pipeline [20]. Finally, we demonstrate the benefits of the improved ontology and annotations for a set of genes differentially expressed in kidney glomeruli affected by the later stages of the disease diabetic nephropathy (DN).

## Methods

### Ontology Development and Annotation

A meeting was held between renal biomedical experts, GO curators and GO editors to determine the correct representation of renal processes (renal development in particular) in the Gene Ontology. Ontology editors added the new terms and reorganized existing renal GO terms accordingly.

For annotation purposes, expression data from the GUDMAP database [8] was used to identify 29 mouse genes expressed in the murine loop of Henle. BLAST [19] was used to identify similar genes, where present, in human, zebrafish, *Xenopus*, chicken and fly. Table 1 lists the UniProtKB accession numbers of the corresponding gene products. Curators from UniProt, Mouse Genome Informatics (MGI), FlyBase and the zebrafish model organism database (ZFIN) [21]–[24] then assigned molecular function, biological process and cellular component GO terms to the gene products, based on experimental evidence in scientific papers.

**Table 1:** UniProtKB accession numbers for 29 homologous proteins using data from *in-situ* hybridisation expression in murine loop of Henle.

	UniProtKB accession number					
Protein name	Mouse	Human	Rat	Chicken	*Xenopus laevis/tropicalis*	*Drosophila melanogaster*
**Tesc**	Q9JKL5	Q96BS2	D3ZTN19	A0AVX7	Q5U554/Q0V9B1	n/a
**Slc23a1**	Q9Z2J0	Q9UHI7	Q9WTW7	B9VMA9	-/B0JZG0	(Q9VH02)
**Ctnnb1**	Q02248	P35222	Q9WU82	O42486	P26233/Q28GC2	P18824
**Lamb1-1**	P02469	P07942	P15800	O57484	Q5XHI6/B3DLV1	(P11046)
**Egr1**	P08046	P18146	P08154	O73691	Q6GQH4/A4II20	n/a
**Pou3f3 (Brn1)**	P31361	P20264	Q63262	Q52HB4 O73861	P70030/A1L0Z1	(P16241)
**Id2**	P41136	Q02363	P41137	O73933	Q9YGL0/Q6PBD7	n/a
**Cdh11**	P55288	P55287	Q9JIW2	O93319	O93264/Q5EAM2	n/a
**Aldh1l1**	Q8R0Y6	O75891	P28037	O93344	Q6GNL7/Q63ZT8	(Q9VIC9)
**Tfap2b**	Q61313	Q92481	P58197	O93346	Q66J14/Q28C75	n/a
**Ttr**	P07309	P02766	P02767	P27731	Q9W649/A4QNN7	n/a
**Ptn**	P63089	P21246	P63090	P32760	P48532/A4IH83	n/a
**Ccnd1**	P25322	P24385	P39948	P55169	P50755/Q6GLD3	n/a
**Irx3**	P81067	P78415	n/a	Q9PUR3	O42261/Q6NVN3	n/a
**Irx2**	P81066	Q9BZI1	n/a	Q9PU52	Q6DCQ1/Q66IK1	n/a
**Irx1**	P81068	P78414	n/a	Q9I9C5	Q9YGK8/Q6F2E3	n/a
**Pax2**	P32114	Q02962	D4ACZ2	Q9PTX1	O57685 O57682/Q28IR6	n/a
**Pax8**	Q00288	Q06710	P51974	n/a	Q9PUK5/A0JMA6	n/a
**Bmp4**	P21275	P12644	Q06826	Q90752	P30885/Q90YD6	n/a
**Cited1**	P97769	Q99966	Q4V8P1	n/a	n/a	n/a
**Cited2**	O35740	Q99967	Q99MA1	Q9DDW4	Q5XGW7/Q6NX30 Q28GT4	n/a
**c-myc**	P01108	P01106	P094169	P01109	P06171/Q6P1T1	n/a
**WT1**	P22561	P19544	P49952	Q9I8A0 Q9I8A1	B7ZSG3 P79958/B5DE03	n/a
**Osr1/Odd1**	Q9WVG7	Q8TAX0	B0K011	E1BWE8	P86413/Q66JF8	P23803
**Osr2**	Q91ZD1	Q8N2R0	Q6AY34	E1BUP0	Q32NK7 Q0IHB8/−	Q9VQS7
**PDGFRB**	P05622	P09619	Q05030	n/a	n/a	n/a
**PDGFRA**	P26618	P16234	P20786	Q9PUF6	P26619/A4IHL2	n/a
**PDGFB**	P31240	P01127	Q05028	Q90W23	Q6DDJ9/B1H1E3 B0BM23	n/a
**PDGFA**	P20033	P04085	P28576	Q90WK2 Q9PUF7	P13698/B0BM23	(Q9VWP6)

Uniprot accession numbers are listed for homologues of the 29 proteins expressed in the murine loop of Henle structure (data provided by the GUDMAP Consortium via www.gudmap.org) as determined by BLAST (run via the uniprot.org website). The *Drosophila* proteins in parentheses are homologous to multiple mammalian proteins. (n/a = not applicable).

Experimentally assigned GO annotations were subsequently transferred to proteins in other species that are similar in sequence; this was performed both manually and electronically. Manually, curators or authors identified the similar targets via sequence similarity search programs such as BLAST [19] or Homologene [25]. Electronically, the experimental annotations acted as a source of projected annotations for orthologous proteins in vertebrate species via the Ensembl Compara automatic annotation pipeline [20].

### GO Term Enrichment Analysis

Two term enrichment tools were used for the analyses; GO-Elite (http://www.genmapp.org/go_elite/) [26], [27] and Ontologizer (http://compbio.charite.de/index.php/ontologizer2.html) [28]; GO term enrichment analysis was performed using annotations to biological process terms only.

We took the gene data set for our reanalysis from the investigation into the differential gene expression in glomeruli from human kidneys with diabetic nephropathy by Baelde *et al*. [29]. The gene identifiers used in this 2004 study were mapped to current UniProtKB accession numbers (Table S1 in File S1). Some of the gene identifiers, for example, D87002, mapped to multiple UniProtKB accessions (Q14390, Q5NV78, Q5NV77) because both ‘reviewed’ and ‘un-reviewed’ sequences in the UniProtKB database cross-referenced to the same gene identifier. However, in such cases only one of the accession numbers for the gene product was curated; usually the reviewed UniProtKB/SwissProt entry or, if all entries were un-reviewed, the longest UniProtKB/TrEMBL sequence. The protein accessions in Table S1, in File S1, constitute the ‘Input’ list for the GO term enrichment analysis.

For the GO-Elite analysis, we used the ORA-pruned analysis with a z-score cut-off of >1.96, the minimum number of changed genes was set at 3 and the permuted p-value cut-off was <0.1. GO-Elite uses the Z-score/hypergeometric statistical method and Benjamini-Hochberg (BH) correction for multiple hypothesis testing [27].

For the Ontologizer analysis, term enrichment was calculated using the parent-child intersection analysis method using a modified Fisher’s exact analysis. The single-step minP procedure of Westfall-Young was applied as a multiple testing correction. Terms were considered significantly enriched if the adjusted p-value was <0.1 [28].

### Data Files

Ontology files were downloaded from: http://cvsweb.geneontology.org/cgi-bin/cvsweb.cgi/go/ontology/gene_ontology.obo.

**Figure 1:**
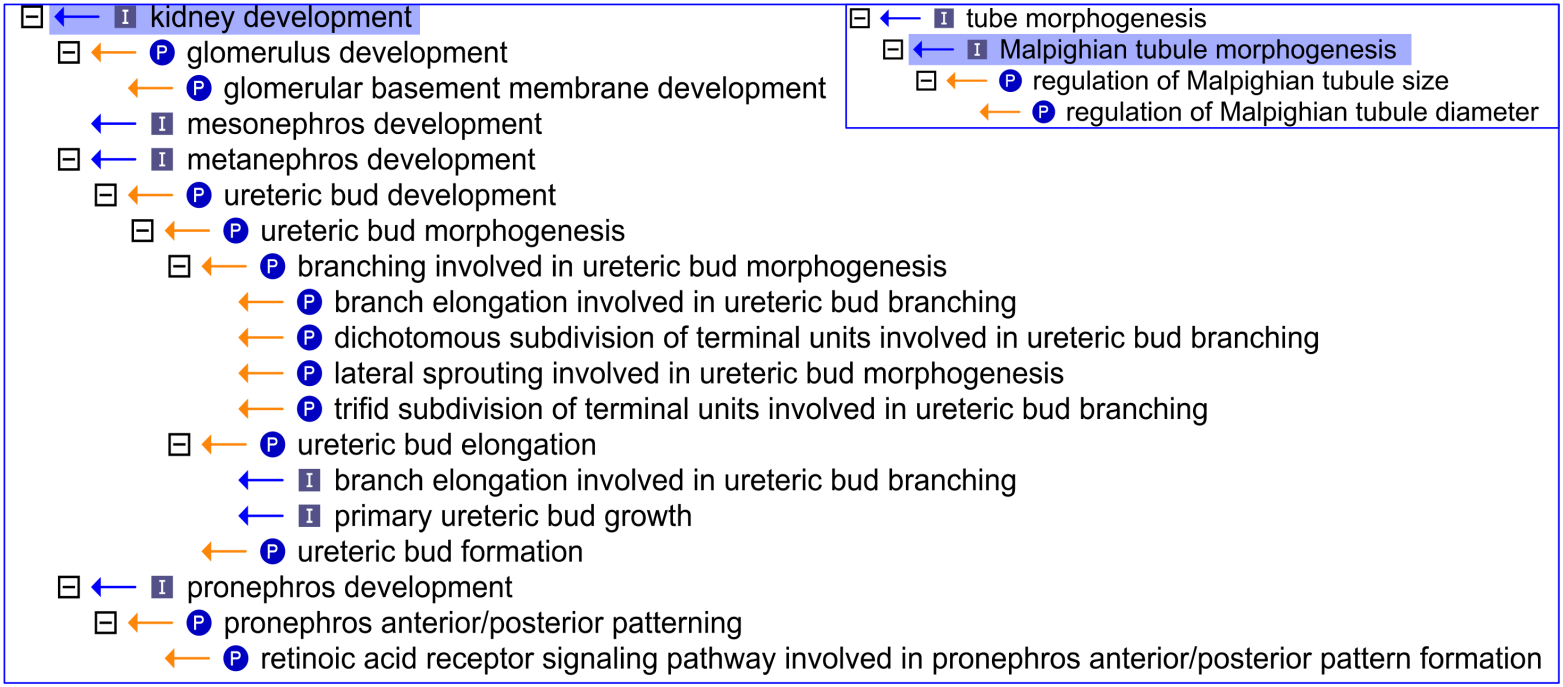
An OBO-Edit ‘Ontology Tree Editor’ view showing the 21 Gene Ontology terms representing renal development before the expansion in this area of the ontology. There were 18 GO terms directly under the ‘kidney development*’* node and 3 terms representing morphogenesis of the insect renal system, the Malpighian tubule (using the Gene Ontology file from November 18^th^ 2009). The [−] icon beside each term denotes no further child terms; (P) denotes a *part_of* relationship; (I) denotes an *is_a* relationship.

**Figure 2:**
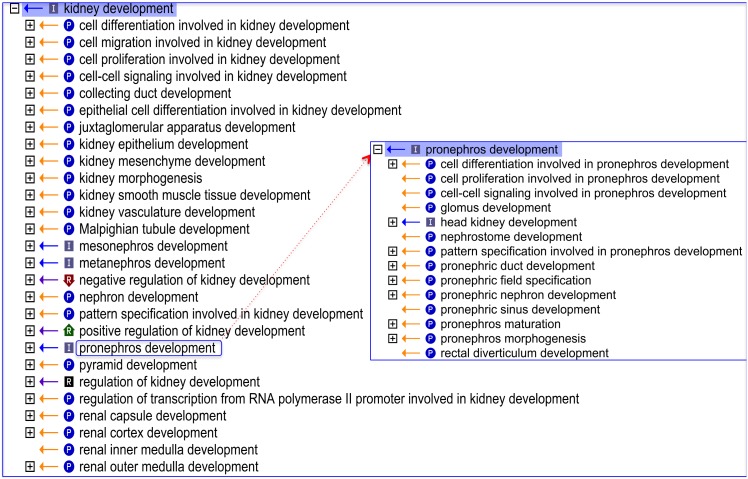
An OBO-Edit ‘Ontology Tree Editor’ view demonstrating the improved Gene Ontology representing ‘kidney development’ after a focused expansion. The Gene Ontology representing kidney development was enriched after a focused expansion with an additional 522 new terms, and as an example (using the Gene Ontology file from March 19^th^ 2012) the expanded node of the ‘*pronephros development*’ term shows it’s immediate child terms. The [+] icon beside each term denotes that there are further child terms that can be viewed; the [−] icon denotes no further child terms; (P) denotes a *part_of* relationship; (I) denotes an *is_a* relationship; (R) denotes a *regulates* relationship.

**Figure 3:**
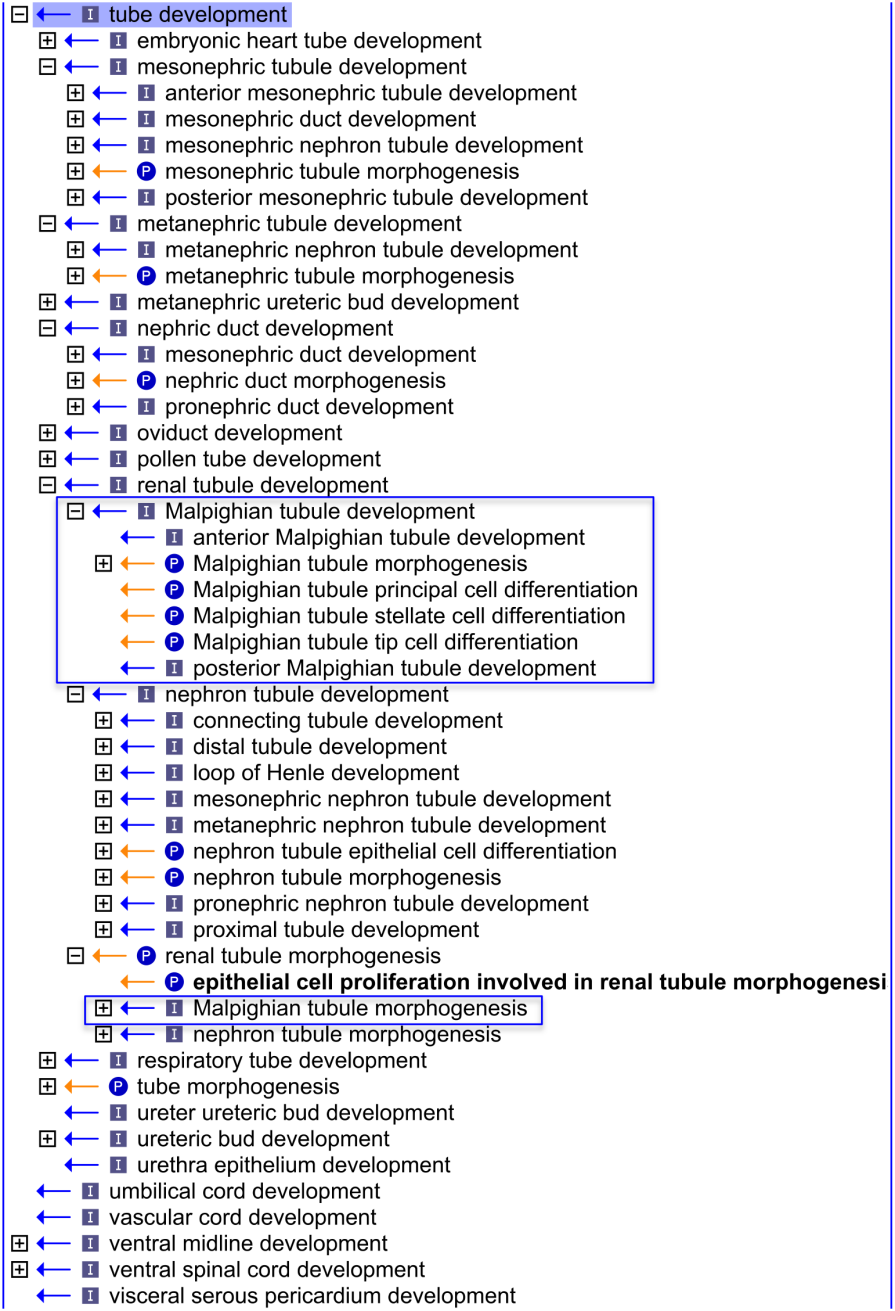
An OBO-Edit ‘Ontology Tree Editor’ view representing similarities in tubule structures and grouped terms describing the development of different types of renal tubules. Similarities are observed in GO terms representing tubule structures and terms are grouped together describing the development of different types of renal tubules including the Malpighian tubule of insects (using the Gene Ontology file from March 19^th^ 2012). The [+] icon beside a term denotes that the node is expandable and has further child/grandchild terms; the [−] icon denotes no further child terms; (P) denotes a *part_of* relationship; (I) denotes an *is_a* relationship.

**Figure 4:**
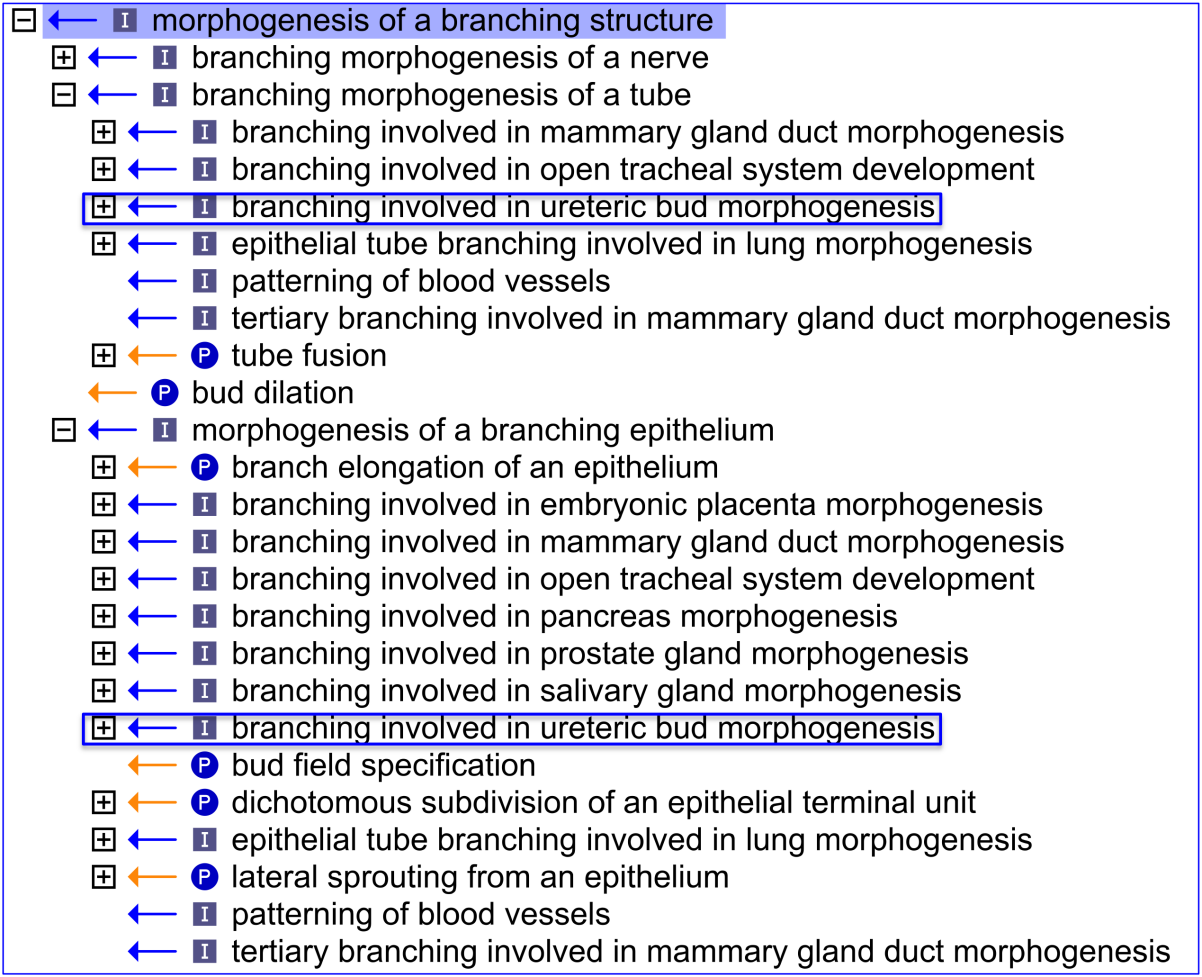
An OBO-Edit ‘Ontology Tree Editor’ view showing the relationship and position of the new GO term ‘*branching involved in ureteric bud morphogenesis*’. By placing the new term ‘*branching involved in ureteric bud morphogenesis*’ as a sub-type of ‘*morphogenesis of a branching structure’*, it puts the renal branching into the context of other types of branching morphogenesis within the Gene Ontology (using the Gene Ontology file from March 19^th^ 2012). The [+] icon beside each term denotes that there are further child terms that can be viewed; the [−] icon denotes no further child terms; (P) denotes a *part_of* relationship; (I) denotes an *is_a* relationship.

Versions of the ontology files downloaded from the above location, used in OBO-Edit for creating Figure 1, were from November 18^th^ 2009 and those for creating Figures 2, 3 and 4 were from March 19^th^ 2012.

Gene Association Files (GO annotation datasets) were downloaded from ftp://ftp.ebi.ac.uk/pub/databases/GO/goa/old/HUMAN/and ftp://ftp.ebi.ac.uk/pub/contrib/goa/ReferenceGenome/20120319/.

File versions used for the 2012, ‘post-annotation dataset’ analysis reported in Tables S2–S9 in File S1 and Tables 2 and 3 were Gene Ontology revision 4.1180 (March 20^th^ 2012) and annotation file ‘gene_association.goa_human.gz’ (March 19^th^ 2012).

**Table 2:** Summary of the number of GO terms significantly enriched in the differentially expressed gene dataset from glomeruli affected by Diabetic Nephropathy (DN) by both Ontologizer and GO-Elite enrichment analysis tools.

Gene set	Number of significantly enriched GO terms (p<0.1)			
	pre-annotation		post-annotation	
	Ontologizer	GO-Elite	Ontologizer	GO-Elite
Up-regulated	42	22	182	139
Down-regulated	48	21	127	85

A summary of the number of GO terms that were significantly enriched (having a p-value of <0.1) in the Baelde groups’ differentially expressed gene dataset from glomeruli affected by DN [29] by both Ontologizer [28] and GO-Elite [27] term enrichment tools, using the pre-annotation (2009) and post-annotation (2012) GO annotation datasets.

**Table 3:** Summary of significantly enriched GO terms from the Ontologizer and GO-Elite analyses that are relevant to kidney development.

GO ID	GO Term Name	Observed in Ontologizer (O);GO-Elite (G)	Rank 2012 n = 127 (O), n = 85 (G)	Rank 2009 n = 48 (O), n = 21 (G)
GO:0032835	glomerulus development	G	1	n/a
GO:0061005	cell differentiation involved in kidney development	G	2	n/a
*GO:0001655*	*urogenital system development*	O	10	14
>GO:2001012	mesenchymal cell differentiation involved in renal system development	O	90	n/a
>GO:0001657	ureteric bud development	O	65	n/a
*GO:0003014*	*renal system process*	O, G	52(O); 25(G)	n/a
>GO:0097205	renal filtration	O	43	n/a
*GO:0001763*	*morphogenesis of a branching structure*	O	64	42
>GO:0048754	branching morphogenesis of an epithelial tube	G	43	n/a

A summary of the significantly enriched GO terms from the Ontologizer [28] and GO-Elite [27] analyses, which are relevant to kidney development, using the pre-annotation (2009; Tables S2–S5 in File S1) and post-annotation datasets (2012; Tables S6–S9, in File S1). Terms in italics indicate parent terms where the descendants are indicated directly underneath as follows: > descendant of term above in italics. Rank refers to the position of the term in the results of the enrichment analyses (see Tables S2–S9 in File S1) where significance of the enriched term has a p-value of <0.1. (n/a = not applicable).

File versions used for the 2009, ‘pre-annotation dataset’ analysis reported in Tables S2–S9 in File S1 and Table 3, were Gene Ontology revision 4.548 (March 5^th^ 2009) and annotation file ‘gene_association.goa_human.72.gz’ (March 5^th^ 2009).

The ‘Background’ list of protein accessions used in the GO term enrichment analyses was obtained from the relevant Gene Association File. For example, the background list for the 2009 analyses was the unique protein accessions in the ‘gene_association.goa_human.72.gz’ file.

Annotation data sets for renal specific gene products and GO terms can be viewed via the QuickGO browser at www.ebi.ac.uk/QuickGO, using either gene product name(s), UniProtKB accession number(s) or GO term(s).

## Results and Discussion

The renal system development node of the GO has been expanded and refined in response to a common need for a computational resource for investigators in this field. The GO Consortium has previously demonstrated success in developing a specific area of the ontology through focus meetings where community experts meet alongside ontology developers to discuss the current knowledge of the biological area of interest and its best representation in the GO [30], [31]. Therefore, this approach was also used in the refinement of renal system development representation in the GO.

### Enhancement of the Gene Ontology for Renal Development

By consulting with renal experts, we have extensively improved the renal development branch of the GO. Prior to this project, the GO contained only 21 terms to describe renal development; 18 terms under and including *‘kidney development’* (GO:0001822) and 3 terms describing development of the insect renal system, the Malpighian tubule (Figure 1). Ultimately this project has resulted in an additional 522 renal development GO terms, including 137 under ‘*metanephros development’* (GO:0001656), 102 terms under ‘*mesonephros development*’ (GO:0001823), 28 terms under ‘*pronephros development*’ (GO:0048793) and 19 terms under ‘*Malpighian tubule development’* (GO:0072002). Figure 2 displays an OBO-Edit Ontology Tree Editor view of a subset of the expanded pronephros development GO terms. Definitions and synonyms of existing terms were also improved. For example, the ambiguity of the word ‘nephrogenesis’ has been addressed by including it as a synonym for both ‘*kidney development*’ (GO:0001822) and ‘*nephron development*’ (GO:0072006), enabling curators to make a decision on which term to choose depending on the evidence they are presented with.

We have made use of existing renal cell and anatomy resources and have ensured that GO terms referencing such structures are also made consistent with those described by the GUDMAP Consortium [8] and the Cell Type Ontology [13]. For example, in creating the new GO term ‘*nephrocyte diaphragm assembly*’ (GO:0036059) we have utilized the cell type ontology term ‘*nephrocyte*’ (CL:0002520). In addition, we extended UBERON [14] to include all the renal structures named within GO terms, and also created cross-references [15] between renal system processes in GO and anatomical structures in UBERON. For example, the term ‘*metanephric mesenchymal cell proliferation involved in metanephros development*’ (GO:0072136) is cross-referenced in UBERON to ‘*metanephric mesenchyme*’ (UBERON:0003220) and ‘*metanephros*’ (UBERON:0000081).

### Representing Anatomical Groupings

We present a framework for the future addition of renal-related ontology terms as knowledge of renal development progresses. This required the careful construction of relationships between the GO terms to place them in context with other cellular and developmental GO processes. One particularly interesting challenge was representing development of renal structures so they could be described as individual structures but also grouped for the purpose of data aggregation. For example, the term ‘renal tubule’ specifies a particular anatomical structure in a renal system and differentiates it from other biological tubules.

The first task was to define a renal system in the context of GO terms. In consultation with the renal experts, we defined ‘*renal system*’ as ‘*a system that maintains fluid balance and contributes to electrolyte balance, acid/base balance and disposal of nitrogenous waste products*’ (see GO:0003014 ‘*renal system process*’)**.** This definition is inclusive of the organs of the vertebrate renal system, as well as the Malpighian tubules of insects, and allows for future incorporation of structures such as the antennal glands of crustaceans. The term ‘*kidney development*’ (GO:0001822) is used to cover the development of the three vertebrate structures; the pronephros, the mesonephros and the metanephros. As these structures all contain tubules that function in the renal system, terms describing the development of each type of renal tubule, such as ‘*Malpighian tubule development*’ (GO:0072002) and ‘*nephron tubule development*’ (GO:0072080) are grouped together under a ‘*renal tubule development*’ (GO:0061326) term (Figure 3). This type of anatomical grouping affords another benefit in allowing comparison of gene products involved in renal tube development with those involved in tube development in other systems including the respiratory system, circulatory system, digestive system and the early embryo. Such comparisons can be used to elucidate common molecular strategies in the development of epithelial tubes.

### Representing Similar Developmental Processes

A critical aspect in understanding the development of a structure is the identification of similar molecular mechanisms that are used repeatedly across development. Grouping renal system processes in the GO with similar processes in other organs can enable the user to predict gene products that may play an important role in renal system development. In the GO, developmental processes are broken down into several categories: morphogenetic mechanisms that shape a structure, signaling mechanisms that allow cells and tissues to communicate, pattern specification mechanisms that lay out the landscape in which cells differentiate, and gene regulatory mechanisms that permit the correct expression of sets of genes responsible for cell differentiation.

Thus we have created terms that describe the morphogenesis of tubes, epithelia and mesenchymal tissues with respect to specific renal structures. For example, ‘*epithelial cell proliferation involved in renal tubule morphogenesis*’ (GO:2001013) and ‘*mesenchymal to epithelial transition involved in metanephric renal vesicle formation*’ (GO:0072285). Moreover, by placing the term ‘*branching involved in ureteric bud morphogenesis*’ (GO:0001658) as a sub-type of ‘*morphogenesis of a branching structure*’ (GO:0001763), renal branching is put into the context of other types of branching morphogenesis in GO (Figure 4).

We have also created terms to describe known inductive events involved in the initial formation of renal structures such as ‘*specification of metanephric proximal tubule identity*’ (GO:0072297) and ‘*anterior/posterior pattern specification involved in pronephros development*’ (GO:0034672).

### Use of the New Renal Development GO Terms in Gene Product Annotation

Following the improved ontology structure for renal system development, curators annotated renal-related gene products. The first annotation targets were the 29 gene products highly expressed in the mouse loop of Henle [8] and hence predicted to play a role in the development and/or physiology of this renal structure. To compare the function of these proteins across species, similar proteins in human, rat, zebrafish, *Drosophila* and *Xenopus* (found via BLAST run on the uniprot.org website) were also annotated. Table 1 lists the UniProtKB accession numbers for these gene products. The loop of Henle plays an important role in creating a concentration gradient in the medulla of the kidney. It is involved in reabsorption of filtered water and ions including sodium, potassium and calcium, and independently regulates both the volume and osmolarity of body fluids. The structure differs considerably between species; there is a definite physical loop of Henle in the mammalian and avian renal systems but this seems to be absent in *Xenopus*. However, homologs of some, but not all, molecular markers (*e.g. cldn8* and *clcnk)* of the mammalian loop of Henle were found to be present in the pronephros of the frog larva [32]. Therefore, annotation of gene products expressed in the loop of Henle structure could provide an insight into how the functions of gene products have evolved. For *Drosophila*, where similar proteins for this set of target proteins were unclear, we attempted to make annotations with all of the 19 new terms that were created to describe Malpighian tubule development; 82 new manual annotations were added for *Drosophila melanogaster* using these terms alone (see section “*Comparison of Renal GO Annotations Across Species*”).

An additional aim of this curation project was to curate the biological roles of human proteins encoded by RNAs previously identified as being differentially expressed in kidney glomeruli affected by late stage diabetic nephropathy (DN) [29] (Table S1 in File S1). The effect of this focused annotation is reported in the later section “*Impact of improved Gene Ontology annotation on data analysis*”.

Although the focus of this project is renal development, this initiative has also expanded curation of the renal physiology and function nodes of GO. Annotations have been made using GO terms that describe aspects of renal processes such as acid-base homeostasis, transmembrane ion (*e.g.* sodium, potassium ion) transport, renal water homeostasis, renal absorption, renal secretion, blood pressure regulation and regulation of urine volume. It should also be noted that improvements to annotations of renal-specific gene products and to the Gene Ontology representing renal processes are an ongoing task, as more biomedical research is published, identifying the role of various existing and newly identified gene products in renal function and development.

In total, this project has manually assigned approximately 9,600 kidney-related GO annotations to 940 distinct UniProtKB protein entries across 66 species and has greatly improved the number and quality of annotations associated with individual proteins. This manual annotation application also benefits orthologous proteins in other species by virtue of the automatic annotation created by Ensembl Compara [20], which projects experimental GO annotation between similar vertebrate species (50,000 electronic annotations were created for over 2,500 UniProtKB entries covering 32 taxa).

The initiative has expanded and improved GO annotation for gene products, as demonstrated by the mouse PAX8 protein (UniProtKB:Q00288). Prior to the start of the annotation project, this protein had been associated with a single renal GO term, ‘*metanephros development’* (GO:0001656). After the focused effort however, it had an extra 32 GO annotations, containing 17 unique renal development terms. The annotation has also introduced more specificity, with terms such as ‘*negative regulation of mesenchymal stem cell apoptotic process involved in metanephric nephron morphogenesis*’ (GO:0072305) and ‘*metanephric distal convoluted tubule development*’ (GO:0072221). The focused annotation of this protein has brought together data from 15 published papers and highlighted the additional involvement of PAX8 in non-renal developmental processes such as ‘*inner ear morphogenesis*’ (GO:0042472) and ‘*thyroid gland development*’ (GO:0030878), thus emphasizing the importance of manual curation for capturing all functional roles of a gene product.

### Comparison of Renal GO Annotations Across Species

The species-neutral nature of GO makes it a powerful tool for cross-species use with the potential to highlight common mechanisms governing renal development. It is unsurprising that GO annotations point to a similar role of renal gene products between human, rat and mouse, but perhaps more interesting is where the similarity of gene roles extends beyond the mammals to frog and fish, and in some cases to fly.

The transcription factors HEY1 and HEYL, LHX1, MECOM, TCF21, WT1 and the Odd-skipped-, PAX- and SOX-family members all have renal GO annotations in multiple species. The Odd-skipped family of proteins (Odd in *Drosophila* and OSR1 and OSR2 in vertebrates) has annotations to renal system development terms in fly (‘*Malpighian tubule morphogenesis’* (GO:0007443)), across fish and frogs (‘*pronephros development’* (GO:0048793)), up to mammals (‘*mesonephros development’* (GO:0001823) and ‘*metanephros development’* (GO:0001656)). OSR1 annotations are more detailed than those for OSR2, given that there is experimental evidence for OSR1 being the earliest marker for intermediated mesoderm, the precursor to the mammalian metanephric kidney [33]. Annotations to OSR2 are more general as they have been assigned from expression patterns and include the terms ‘*metanephros development’* (GO:0001656) and ‘*mesonephros development*’ (GO:0001823). This may reflect the fact that the role of OSR2 in mammalian kidney development is less clear than for OSR1, or that despite renal expression patterns, mouse OSR2 may not be required for mouse kidney development [34]. Conversely, in *Xenopus* and zebrafish both Osr1 and Osr2 have been demonstrated to have essential roles in pronephros development [35].

The PAX transcription factors are similarly known to be important regulators of kidney development [36], with PAX2 defects resulting in renal diseases including renal hypodysplasia [37]. GO annotations in organisms including *Xenopus*, zebrafish, mouse and human confirm a role for the PAX2 and PAX8 transcription factors in processes including ‘*pronephric field specification’* (GO:0039003) [38], ‘*regulation of kidney size*’ (GO:0035564) [39] and ‘*branching involved in ureteric bud morphogenesis’* (GO:0001658) [40].

The ‘NOT’ qualifier has proven useful in the renal annotation of members of *Iroquois* protein family. GO annotations point to at least the IRX3 transcription factor being involved in directing nephron identity. *Xenopus laevis* Irx1-a and Irx3 are annotated to ‘*specification of pronephric tubule identity*’ (GO:0039005) [41], [42] whilst mouse IRX2 and IRX3 have annotations to ‘*specification of loop of Henle identity*’ (GO:0072086) [42]. With the ‘NOT’ qualifier in place, annotations to *Xenopus* Irx4-A and Irx5 proteins state that these family members do not have a role in frog pronephros development, thus indicating divergence in the functions of this protein family.

Many signaling cascades trigger the activation of transcription factors and we identify signaling pathways involved in the development of renal tubules in multiple species. GO annotations point to a role for WNT family members in renal development; *Drosophila* Wingless (Wg) has Malpighian tubule GO annotations, with renal annotations continuing for vertebrate WNT proteins, most notably ‘*ureteric bud development*’ (GO:0001657) in human and mouse, and ‘*glomus development*’ (GO;0072013) in *Xenopus.* Thus, despite the morphological differences in kidney structure between organisms as diverse as frogs and humans, the nephron units show similar functions and the genes regulating development of these units show functional similarities.

Members of the bone morphogenetic protein (BMP) family are important signaling molecules, and GO annotations point to a critical role for BMP signaling in the development of renal structures across species. Sonic hedgehog (SHH) and its receptor, PATCHED (PTCH1) have annotations to broad kidney development terms, from fish to mouse and human. *Drosophila* Decapentaplegic (Dpp), together with the intracellular SMAD proteins Mothers against Dpp (Mad) and Daughters against Dpp (Dad, an inhibitory SMAD) all have annotations to ‘*BMP signaling pathway involved in Malpighian tubule cell chemotaxis’* (GO:0061353) and related terms. Similarly, in vertebrates including chicken, mouse and human, BMP2 and BMP4 proteins have annotations to a range of kidney development terms including ‘*ureteric bud development*’ (GO:0001657) and ‘*ureter epithelial cell differentiation*’ (GO:0072192). Other members of the BMP signaling pathway also show renal annotations. The secreted Dpp/BMP-inhibitors Short gastrulation (Sog) in *Drosophila* and Chordin in *Xenopus,* have annotations to ‘*posterior Malpighian tubule development’* (GO:0061328) and ‘*pronephros development’* (GO:0048793), respectively. Likewise, the BMP antagonist GREM1 shows ‘*pronephros development*’ (GO:0048793) annotations in *Xenopus* and ‘*ureteric bud morphogenesis’* (GO:0060675) and ‘*metanephros morphogenesis*’ (GO:0003338) annotations in mouse, thus providing further hints for a comparable BMP signaling pathway in renal tubule development across species.

In summary, this annotation exercise reveals interesting comparisons of renal development across species and has confirmed that although the kidney structures themselves differ between insects, non-mammalian vertebrates and mammals, some of the associated gene products and pathways show similar roles in renal development.

### Impact of Improved Gene Ontology Annotation on Data Analysis

To test the value of increasing the depth and coverage of GO annotation for renal-related proteins on interpretation of experiments, we compared the annotation dataset from immediately prior to our focused annotation project (March 2009, and referred to hereafter as the ‘pre-annotation dataset’) with the dataset from the end of the project (March 2012, and referred to hereafter as the ‘post-annotation dataset’). To this end, we fully annotated the biological roles of a set of human proteins that were initially identified in a study of genes differentially expressed in glomeruli of kidneys affected by the later stages of diabetic nephropathy (DN), as reported by Baelde *et al.* [29]. We chose to test the improvement in the annotation dataset by performing a term enrichment analysis on the differentially expressed gene products identified in the original study, as this is an efficient way to get an overview of the annotations for a set of genes without losing the specificity of the added GO terms, and can be used to directly compare the results obtained by Baelde *et al*. [29] in their original analysis.

DN is characterized by increased levels of albumin in the urine (albuminuria) associated with a combination of altered glomerular hemodynamics and a thickened glomerular basement membrane. In the early stages of DN there is a phase of overgrowth caused by angiogenesis and endothelial hyperproliferation, which results in a corresponding increase in the glomerular filtration rate. This is followed, in the later stages, by capillary loss and fibrosis, progressing to renal failure. Hence, processes involved in the progression of DN include altered endothelial cell turnover [43]–[45], epithelial-to-mesenchymal transition involving the cytokine transforming growth factor-beta1 (TGFβ1) [46] and altered vascular growth factor signaling [47]. The diabetic kidneys used by Baelde *et al*. [29] contained “nodular glomerulosclerosis and arteriolar hyalinosis”, hence they were in the later stages of the disease.

Baelde *et al.* [29] performed term enrichment analysis on the identified up- and down-regulated genes and reported the appearance of GO terms that are noticeably high-level, less-specific terms such as *‘intracellular signaling transduction’* (GO:0035556), ‘*negative regulation of cell proliferation’* (GO:0008285), *‘homeostatic process’* (GO:0042592) and *‘nucleobase-containing compound metabolic process’* (GO:0006139). Unfortunately, these types of terms convey little information about the specific role of a gene product in DN and there was evidence that the gene products were lacking sufficient functional annotation. The GO term enrichment analysis reported by Baelde *et al.* in 2004 [29] was performed using MappFinder [26], however this is no longer supported, so we used its sister tool, GO-Elite [27] to perform the reanalysis of the datasets. In a previous study [48], we demonstrated the need to use a variety of GO analysis tools to ensure a balanced interpretation of the dataset, therefore we also used Ontologizer [28].

### Overall Observations from the Analysis

The analysis was performed separately on up- and down-regulated genes since this distinction was made in the original analysis [29]. Full term enrichment results for both up- and down-regulated genes using both GO-Elite and Ontologizer are available as Tables S2–S9 in File S1.

Table 2 summarizes the number of terms significantly enriched by both Ontologizer and GO-Elite term enrichment tools using the GO annotation datasets from before and after the annotation focus. The most striking observation is that, in the output from both tools, there are significantly more enriched terms using the post-annotation dataset, compared to using the pre-annotation dataset (significance cut-off values are reported in the ‘Methods’ section). In general, the terms that were significantly enriched using the post-annotation dataset were not enriched using the pre-annotation dataset, indicating that focused GO annotation using both existing and the new terms created during this initiative has had a great impact on the interpretation of this analysis.

#### Enrichment of terms related to kidney development

A significant observation from our analysis was the appearance of some of the new renal development GO terms created by our ontology improvements, summarized in Table 3. For example, *‘mesenchymal cell differentiation involved in renal system development’* (GO:2001012) (Table S9 in File S1) and *‘cell differentiation involved in kidney development’* (GO:0061005) (Table S8 in File S1). The impact of the focused annotation was also recognized by the appearance of some older terms that were available at the time of the original 2004 study, but which had not been associated with the study proteins at that time, for example, ‘*branching morphogenesis of an epithelial tube’* (GO:0048754) (Table S8 in File S1), ‘*renal system process’* (GO:0003014) (Tables S6–S9 in File S1), *‘glomerulus development’* (GO:0032835) (Table S8 in File S1) and *‘ureteric bud development’* (GO:0001657) (Table S9 in File S1). This was likely due to lack of experimental data and/or lack of curation.

#### Enrichment of terms not specific to kidney development

We noted also the appearance of GO terms describing biological processes that are not specific to kidney development, but are still relevant to DN. The importance of extracellular matrix proteins in the expansion of the mesangial matrix and thickening of basement membranes that occurs in DN has already been reported by Abrass [49]. Although no related terms were reported in the original analysis by Baelde *et al.* [29], in the post-annotation analysis we see the terms ‘*extracellular matrix organization*’ (GO:0030198) (Table S6 in File S1) and ‘*basement membrane organization’* (GO:0071711) (Table S7 in File S1) significantly enriched. ‘*Extracellular matrix organization*’ (GO:0030198) was available for annotation in 2004 but only 7 human gene products had been associated with it, none of which were from the Baelde study set. Basement membrane-related process terms were not added to the ontology until 2008 and so were unavailable for use at the time of the Baelde study. Together, this demonstrates ongoing improvements to the Gene Ontology and GO annotations since 2004, as well as this focused annotation project, contributed to the improved results for the post-annotation dataset.

DN can occur due to longstanding diabetes mellitus, a metabolic disease in which an individual has high blood sugar, either because the body does not produce enough insulin, or because cells do not respond to the insulin that is produced [50], [51]. Therefore it is encouraging to see the appearance of *‘positive regulation of insulin secretion involved in cellular response to glucose stimulus’* (GO:0035774) and *‘response to insulin’* (GO:0032868) (Table S6 in File S1) following the focused annotation.

There is increasing evidence that there is an inflammatory aspect to DN [52] and this is reflected in the terms that were significantly enriched in the post-annotation analysis, such as ‘*inflammatory response*’ (GO:0006954) (Tables S6 and S8 in File S1), *‘regulation of cytokine secretion’* (GO:0050707) (Table S6 in File S1), ‘*immune system process*’ (GO:0002376) and the regulation thereof (Tables S7–S9 in File S1), *‘T cell mediated immunity’* (GO:0002456) (Table S7 in File S1), ‘*interleukin-10 production*’ (GO:0032613) (Table S7 in File S1) and *‘myeloid leukocyte activation’* (GO:0002274) (Table S6 in File S1). The only terms relating to immunity or inflammation in the pre-annotation dataset were ‘*T cell homeostatic proliferation’* (GO:0001777), *‘lymphocyte apoptotic process’* (GO:0070227) and ‘*immune system process’* (GO:0002376) (Tables S2 and S3 in File S1).

In general, we see only a small number of differences in GO terms enriched for the up-regulated genes versus the down-regulated genes. One difference was the presence of terms describing signaling via transforming growth factor-beta and vascular endothelial growth factor, which are known to mediate aspects of DN. The TGF-beta signaling pathway mediates apoptosis of endothelial cells during normal maturation of glomerular capillaries [43]. However, in the later stages of diabetic glomerular disease, TGF-beta1 induces fibrosis and enhances capillary loss. Additionally, VEGF is an important mediator of endothelial cell proliferation and one of the hallmarks of the later stages of DN is mesangial proliferation and reduced endothelial proliferation as a result of VEGF down-regulation. During the early stages of DN, VEGF is up-regulated and there is active angiogenesis with endothelial hyperproliferation. As the disease progresses, VEGF signaling is decreased and there is a loss of capillary action in the later stages of DN [47]. Consequently, we observed terms involving the TGF-beta signaling pathway in the analysis of the up-regulated set of genes, including *‘response to growth factor’* (GO:0070848; a parent of *‘response to TGF beta’* (GO:0071559), which was also present), *‘TGF beta production’* (GO:0071604) and *‘endothelial cell apoptotic process’* (GO:0072577) (Table S7 in File S1). Whereas in the analysis of the down-regulated genes we observed terms involving the VEGF pathway, such as *‘vascular endothelial growth factor* (*VEGF) receptor signaling pathway’* (GO:0048010) (Tables S8 and S9 in File S1), *‘cellular response to VEGF stimulus’* (GO:0035924) and *‘endothelial cell proliferation’* (GO:0001935) (Table S9 in File S1). Only one of these terms, *‘VEGF receptor signaling pathway’* (GO:0048010) was present in the analyses using the pre-annotation dataset (Table S5 in File S1). These results suggest that genes influencing endothelial cell proliferation may be down-regulated in DN, whereas genes influencing endothelial cell apoptosis may be up-regulated.

A second difference between the up- and down-regulated gene sets was the appearance of nitric oxide-type terms. One of the roles of nitric oxide is to help control blood pressure in the kidney, so the presence of these terms suggests that nitric oxide may play a role in the progression of DN. It has recently been shown that nitric oxide is reduced in diseased kidneys [53], however the increased severity of endothelial dysfunction in DN has been demonstrated in a mouse diabetic model, which has an endothelial nitric oxide (eNO) synthase gene knock out [54]. We observed terms such as ‘*nitric oxide transport’* (GO:0030185) (Table S7 in File S1) and ‘*regulation of nitric oxide biosynthetic process’* (GO:0045428) (Table S6 in File S1), only in the analysis of the up-regulated set of genes using the post-annotation dataset.

It should be noted at this point that researchers can choose from many freely available GO analysis tools to interpret their datasets. However, each tool will give a different interpretation of the dataset, as demonstrated in this paper, with the results from GO-Elite and Ontologizer. This is usually due to the different analysis and correction methods, statistics, filters and versions of the ontology and annotation files that each tool integrates in order to analyze the gene lists [55]. Nevertheless, our analyses using the new set of ontology terms and annotations from the focused annotation initiative, contained more specific and up-to-date results that are in line with current knowledge about DN. This has demonstrated that combining the published knowledge about this distinct set of gene products together with the improved ontology terms has greatly enhanced the interpretation of the significance of the differentially expressed genes in DN, thus allowing us to easily highlight the molecular processes involved in this disease.

## Conclusion

We have improved the structure and content of the Gene Ontology in the area of renal development, providing a single, freely available resource that can be utilized beneficially by the biomedical research community. By way of example, we demonstrate that comprehensive annotation of a discrete set of proteins, using the new ontology structure, can significantly influence the interpretation of both small and large-scale data analyses. Our work has not only improved functional annotation for this relatively small set of proteins; during the course of this project we have added GO annotations to almost 1000 proteins from over 60 species. We have also laid the groundwork for annotation of further gene products that are outside the scope of this project; since this curation project ended in March 2012, the 522 new GO terms have been used to create almost 29,000 annotations to 12,800 distinct proteins, by manual and electronic curation methods.

This paper highlights the importance for both continued development of the Gene Ontology and comprehensive GO annotation of proteins within this resource, can enable researchers to gain improved biological insights into their particular proteins of interest and consequently guide new investigations into understanding the mechanisms of, and propose new treatments for, renal diseases.

## Supporting Information

File S1 File S1. includes Tables S1 to S9 presented in separate tabs of an Excel spreadsheet, representing the input gene product list and the output of the GO term enrichment analyses from GO-Elite and Ontologizer Enrichment tools using the 2009 and 2012 annotation and Gene Ontology datasets for the differentially expressed gene products in the Baelde 2004 study. A description tab entitled ‘Tables S2–S9 Description’, has been included, defining the output from the GO-Elite and Ontologizer GO term enrichment tools presented in Tables S2–S9. **Table S1.** **Input protein list. Mapping of the gene product identifiers from the Baelde 2004 study to UniProtKB accession numbers.** Most of the differentially expressed gene products in DN glomeruli were mapped to a UniProtKB accession number and those that could not be mapped were not annotated and are not included in the table. **Table S2.** **GO-Elite analysis on up-regulated proteins in DN glomeruli, using the 2009 Gene Ontology and annotation sets.** Results from the GO-Elite enrichment analysis tool on the up-regulated proteins from the Baelde using the Gene Ontology and Annotation files from March 2009; showing significantly enriched GO terms. The boldface terms are referred to in the manuscript text. The “Study” column shows the number of proteins in the input list with an annotation to the given term. The “Population” column shows the number of proteins in the background list with an annotation to the given term. **Table S3.** **Ontologizer analysis on up-regulated proteins in DN glomeruli, using the 2009 Gene Ontology and annotation sets.** Results from the Ontologizer enrichment analysis tool on the up-regulated proteins from the Baelde list, using the Gene Ontology and Annotation files from March 2009; showing significantly enriched GO terms. The boldface terms are referred to in the manuscript text. The “Population” column shows the number of proteins in the background list with an annotation to the given term. The “Study” column shows the number of proteins in the input list with an annotation to the given term. **Table S4.** **GO-Elite analysis on down-regulated proteins in DN glomeruli, using the 2009 Gene Ontology and annotation sets.** Results from the GO-Elite enrichment analysis tool on the down-regulated proteins from the Baelde list, using the Gene Ontology and Annotation files from March 2009; showing significantly enriched GO terms. The “Study” column shows the number of proteins in the input list with an annotation to the given term. The “Population” column shows the number of proteins in the background list with an annotation to the given term. **Table S5.** **Ontologizer analysis on down-regulated proteins in DN glomeruli, using the 2009 Gene Ontology and annotation sets.** Results from the Ontologizer enrichment analysis tool on the down-regulated proteins from the Baelde list, using the Gene Ontology and Annotation files from March 2009; showing significantly enriched GO terms. The boldface terms are referred to in the manuscript text. The “Population” column shows the number of proteins in the background list with an annotation to the given term. The “Study” column shows the number of proteins in the input list with an annotation to the given term. **Table S6.** **GO-Elite analysis on up-regulated proteins in DN glomeruli, using the 2012 Gene Ontology and annotation sets.** Results from the GO-Elite enrichment analysis tool on the up-regulated proteins from the Baelde list, using the Gene Ontology and Annotation files from March 2012; showing significantly enriched GO terms. The boldface terms are referred to in the manuscript text and the italicized boldface indicates new terms created during the Renal GO Annotation Initiative. The “Study” column shows the number of proteins in the input list with an annotation to the given term. The “Population” column shows the number of proteins in the background list with an annotation to the given term. **Table S7.** **Ontologizer analysis on up-regulated proteins in DN glomeruli, using the 2012 Gene Ontology and annotation sets.** Results from the Ontologizer enrichment analysis tool on the up-regulated proteins from the Baelde list, using the Gene Ontology and Annotation files from March 2012; showing significantly enriched GO terms. The boldface terms are referred to in the manuscript text and the italicized boldface indicates new terms created during the Renal GO Annotation Initiative. The “Population” column shows the number of proteins in the background list with an annotation to the given term. The “Study” column shows the number of proteins in the input list with an annotation to the given term. **Table S8.** **GO-Elite analysis on down-regulated proteins in DN glomeruli, using the 2012 Gene Ontology and annotation sets.** Results from the GO-Elite enrichment analysis tool on the down-regulated proteins from the Baelde list, using the Gene Ontology and Annotation files from March 2012; showing significantly enriched GO terms. The boldface terms are referred to in the manuscript text and the italicized boldface indicates new terms created during the Renal GO Annotation Initiative. The “Study” column shows the number of proteins in the input list with an annotation to the given term. The “Population” column shows the number of proteins in the background list with an annotation to the given term. **Table S9.** **Ontologizer analysis on down-regulated proteins in DN glomeruli, using the 2012 Gene Ontology and annotation sets.** Results from the Ontologizer enrichment analysis tool on the down-regulated proteins from the Baelde list, using the Gene Ontology and Annotation files from March 2012; showing significantly enriched GO terms. The boldface terms are referred to in the manuscript text and the italicized boldface indicates new terms created during the Renal GO Annotation Initiative. The “Population” column shows the number of proteins in the background list with an annotation to the given term. The “Study” column shows the number of proteins in the input list with an annotation to the given term.
(XLSX)
